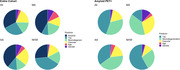# The importance of AT(N)‐V imaging biomarkers differs in diverse populations

**DOI:** 10.1002/alz.093696

**Published:** 2025-01-09

**Authors:** Karin L. Meeker

**Affiliations:** ^1^ Washington University in St. Louis School of Medicine, St. Louis, MO USA

## Abstract

**Background:**

Investigations into the amyloid, tau, and neurodegeneration (ATN) framework, which is used to stage Alzheimer disease (AD) and advance clinical trials, typically consist of clinic‐based non‐Hispanic White (NHW) populations. The present study sought to cross‐sectionally characterize AT(N) and vascular (V) imaging markers (i.e., amyloid and tau positron emission tomography [PET], AD cortical thickness signature, and white matter hyperintensities), and determine the relative importance of each marker in predicting cognitive status (Clinical Dementia Rating Scale Sum of Boxes) in a large ethnically and racially diverse sample.

**Method:**

Data were obtained from the community‐based Health and Aging Brain Study – Health Disparities (HABS‐HD) and included Mexican Americans (MAs, n=669), African Americans (AAs, n=747), and NHWs (n=768). Group differences in AT(N)‐V biomarkers were assessed using Kruskal Wallis ANOVAs. Age and gender were included as covariates. Dominance analysis determined the relative ‘importance’ of each biomarker in predicting cognitive status in the entire cohort and separately for each group. All analyses were performed for the entire cohort, and for individuals on the AD trajectory (i.e., amyloid PET+).

**Result:**

Across the entire cohort, amyloid was significantly greater in AAs compared to NHWs (p’s < 0.01). Tau and neurodegeneration were greater in MAs and AAs compared to NHWs (p’s < 0.01). White matter hyperintensities were greater in AAs and NHWs compared to MAs (p’s < 0.01). There were no significant group differences in AT(N)‐V measures in individuals who were amyloid PET+ (p’s > 0.05). In the entire cohort, A was the most important biomarker (as evidenced by greater R2 values; see Figure 1) in predicting cognitive status. In MAs and AAs, A was also the most important while in NHWs, T was the most important. When constraining to amyloid PET+ individuals, T was most important across all individuals and in NHWs while N was most important in MAs and AAs.

**Conclusion:**

These results are important for evaluation of the new AT(N) criteria and suggest that the magnitude and overall contributions of AT(N)‐V imaging biomarkers in predicting cognitive outcomes may vary among ethno‐racial groups. Possible differential effects of AD biomarkers should be considered in research, clinical trials, and response to treatment.